# P-1327. Enhancing Sexual Health Access for Hispanic and Latino Men in the Southern U.S through a Bilingual Peer-Navigation Campaign

**DOI:** 10.1093/ofid/ofae631.1505

**Published:** 2025-01-29

**Authors:** Carlos S Saldana, Raul Perez, Lily Bonadonna, Jane Y Scott, Karina Gonzalez, Joshua O’neal, David P Holland, Eric Rangel, Brad Cooper, Phyllis Mwaura, Alana Sulka, Dorian Freeman, Audrey Arona, Karie Reed, Pete Clark, Yazmin Silva, Sebastian Gonzalez, Mario Zuluaga, Valeria D Cantos

**Affiliations:** Emory University School of Medicine, Cleveland, Ohio; StopHIVATL.org, Atlanta, Georgia; Emory University School of Medicine Department of Internal Medicine, ATLANTA, Georgia; Emory University School of Medicine, Fulton County Board of Health, Atlanta, Georgia; Rollins School of Public Health, Atlanta, Georgia; Fulton County Board of Health, Atlanta, Georgia; Mercy Care Health Centers, Atlanta, Georgia; Latino LinQ, Atlanta, Georgia; Bear Paw Partners, Atlanta, Georgia; GNR Public Health (Gwinnett, Newton, and Rockdale County Health Departments), Gwinnett, Georgia; GNR Public Health (Gwinnett, Newton, and Rockdale County Health Departments), Gwinnett, Georgia; Gwinnett, Newton & Rockdale County Health Departments, Lawrenceville, Georgia; GNR Public Health (Gwinnett, Newton, and Rockdale County Health Departments), Gwinnett, Georgia; Cobb-Douglas Public Health, Atlanta, Georgia; Georgia Harm Reduction Coalition, Stone Mountain, Georgia; Georgia Harm Reduction Coalition, Stone Mountain, Georgia; MAZDI Agency, Atlanta, Georgia; MAZDI Agency, Atlanta, Georgia; Emory University School of Medicine, Atlanta, GA

## Abstract

**Background:**

Hispanic and Latino Gay and Bisexual Men (HLGBM) are disproportionately impacted by HIV and face structural barriers in accessing sexual health services. To address these barriers, we developed an online sexual health outreach campaign linked to bilingual peer-navigation services in metro Atlanta.

**Figure 1:**
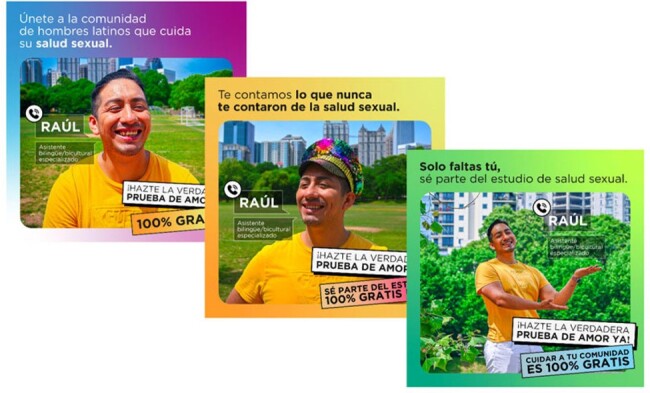
illustrates examples from our sexual health campaign, featuring our trained bilingual navigator. The campaign emphasized the availability of free or low-cost sexual health services, aiming to increase awareness and accessibility among the community.

**Methods:**

In partnership with local organizations, health departments, and Latino-focused advertising agencies, we developed an online outreach campaign in Spanish to increase awareness of sexual health services among HLGBM. This campaign also connected individuals with a trained bilingual Latino peer navigator for service access **(Figure 1).** The campaign was piloted from May 22 to August 21, 2023 (Pilot Phase), it was later adopted by a local health department starting September 1, 2023. We systematically recorded data including the number of individuals contacting the peer navigator, city locations, weekly contacts, referral types, and self-reported outcomes until February 29, 2024 (Post-implementation Phase).

**Figure 2:**
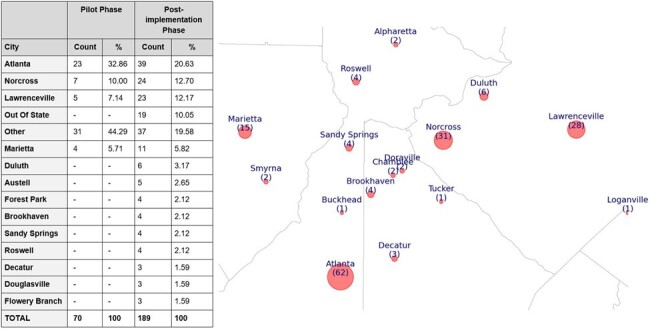
lists the cities of individuals who have contacted the navigator. Cities with two or fewer individuals are grouped under "Other." "Out of State" includes contacts primarily from Tennessee, Alabama, North Carolina, and South Carolina. In the figure, red circles represent the locations from which contacts were made. The size of each circle is proportional to the number of contacts originating from that area.

**Results:**

A total of 259 individuals responded to the campaign by contacting the navigator – 70 during the pilot phase and 189 during the post-implementation phase. The campaign reached individuals from several locations with high Hispanic/Latino populations in the Atlanta area **(Figure 2).** HIV/STI testing and HIV Pre-Exposure Prophylaxis (PrEP) were the most requested services. Participants initially requesting testing HIV/STI only were also provided with PrEP education and referrals. Importantly, a total of 28 participants requested linkage or re-engagement to HIV care **(Table 1)**. During the pilot, 57% of all enrolled participants were successfully linked to services, while 85% did so post-implementation, reaching a higher linkage proportion (**Table 1)**. On average 6 (range 1-19) individuals contacted the navigator per week (**Figure 3)**.

**Table 1:**
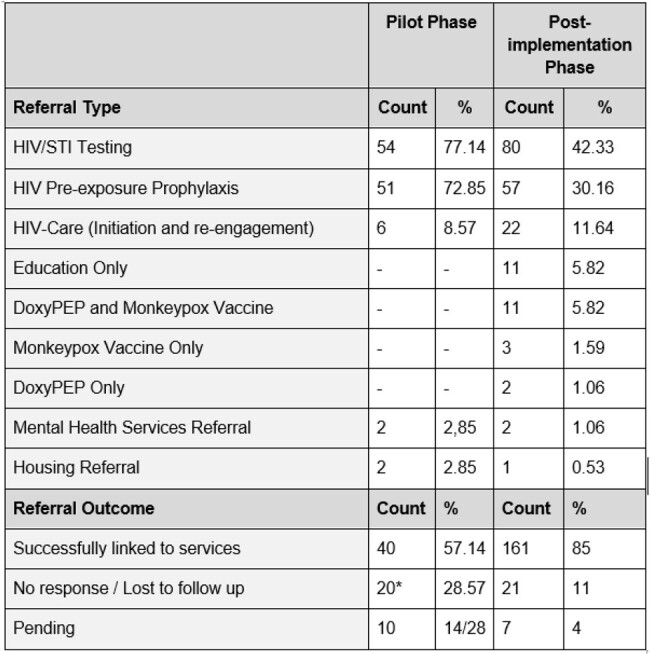
categorizes the types of services requested by individuals through the navigator. Services are grouped by demand frequency. Referral outcomes as reported by the client or the referral agency. “Completed” refers to cases where the patient received the intended service or referral. “Lost to Follow Up” indicates cases where the patient did not continue communication with the navigator, and “Pending” refers to ongoing referrals that have not yet concluded. *Only 50/70 patients in the pilot phase were followed for completion of services.

**Conclusion:**

Our campaign and navigation program successfully reached HLGBM and linked them to local sexual health services. The pilot’s success led to the adoption of the campaign and navigation system by a local health department, with early data suggesting continued community engagement and service linkage, affirming the impact of culturally tailored public health interventions.

**Figure 3:**
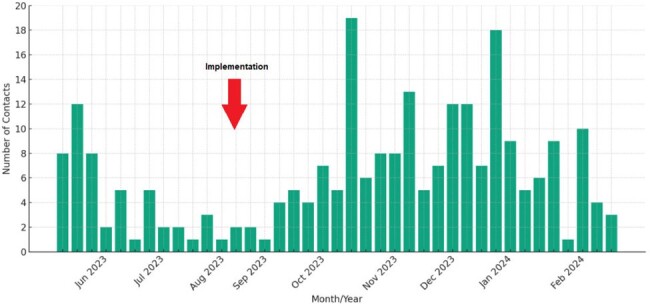
Displays the weekly contacts to the navigator during the pilot and post implementation phase. The arrow indicates when the navigator program was adopted by the Fulton County Board of Health. Data reflects sustained levels of interaction between individuals and the navigator throughout both phases, indicating robust engagement over time.

**Disclosures:**

**All Authors**: No reported disclosures

